# Juvenile dermatomyositis complications: navigating gastrointestinal perforations and treatment challenges, a case report

**DOI:** 10.3389/fped.2024.1419355

**Published:** 2024-07-12

**Authors:** Chen Xiangyuan, Zeng Xiaoling, Sun Guangchao, Zeng Huasong, Liu Dexin

**Affiliations:** ^1^ Department of Allergy, Immunology and Rheumatology, Guangzhou Women and Children’s Medical Center, Guangzhou Medical University, Guangzhou, China; ^2^ Department of Allergy, Immunology and Rheumatology, Liuzhou Hospital, Guangzhou Women and Children’s Medical Center, Liuzhou, China

**Keywords:** Juvenile dermatomyositis, anti-nuclear matrix protein 2, gastrointestinal perforation, Janus kinase inhibitors, case report

## Abstract

Juvenile dermatomyositis (JDM) is a rare autoimmune disorder with multi-system involvement, often presenting with a heliotrope rash, Gottron's papules, and proximal muscle weakness. JDM patients with anti-nuclear matrix protein 2 (anti-NXP2) positivity tend to have more severe manifestations, including a higher risk of gastrointestinal (GI) complications such as dysphagia, intestinal motility changes, edema, malabsorption, ulcers, and perforations. These complications are associated with poor outcomes and high mortality rates, particularly in patients with anti-NXP2 positivity. A case is presented of a 12-year-old girl with JDM who developed multiple GI perforations after being treated with high-dose methylprednisolone. Despite multiple surgical attempts, the patient experienced continued leakage and new perforations. The treatment approach was shifted to include jejunostomy, plasma exchanges, fresh frozen plasma support, and tofacitinib, leading to gradual improvement in muscle strength and reduction in inflammation. GI involvement in JDM is a significant concern due to its association with poor prognosis and high mortality. The use of high-dose glucocorticoids must be carefully considered in JDM patients with GI involvement, as they may contribute to the development of perforations and complicate treatment. A combination of plasma exchange, fresh frozen plasma support, low-dose glucocorticoids, and Janus kinase inhibitors may offer a safer treatment strategy for managing refractory JDM with GI complications. The case highlights the importance of a multidisciplinary approach to treatment and the need for further research to determine the necessity of high-dose glucocorticoid therapy following GI involvement in JDM.

## Introduction

Juvenile dermatomyositis (JDM) is a rare autoimmune disorder characterized by multi-system involvement, which is a vasculopathy characterized by muscle inflammation (1). JDM patients who test positive for anti-nuclear matrix protein 2 (anti-NXP2) often exhibit more severe clinical manifestations, including gastrointestinal (GI) involvement, calcinosis, edema, skin ulcers, and a poorer prognosis (2, 3). Approximately 22%–37% of JDM patients may develop GI complications such as dysphagia, altered intestinal motility, intestinal wall edema, malabsorption, ulcers, and perforations, which can be attributed to intestinal vasculitis and vascular damage (4). Severe GI symptoms are associated with increased mortality rates and are particularly prevalent among JDM patients with anti-NXP2 positivity (2, 5). The incidence of GI manifestations in anti-NXP2 positive JDM patients is reported to be 16.13% (5 out of 31 patients), with the mortality rate due to GI perforation standing at 38% (8 out of 21 patients) (5). JDM with GI tract involvement and perforation is often associated with the disease activity of JDM or the use of high-dose steroid (5–7). Post-perforation treatment for JDM patients includes not only surgical intervention but also a range of immunosuppressive therapies, glucocorticoids, biologic agents, Janus kinase inhibitors (JAKi), intravenous immunoglobulin (IVIG), and plasma exchange (8–12). However, due to the severity of the disease, complications such as severe infections or exacerbation of the primary condition can still lead to a poor prognosis.

In this report, we present a case of JDM with multiple high-grade GI perforations after high-dose steroid. The patient underwent three surgical repairs, all of which resulted in continued leakage and new perforations were found. Ultimately, the patient's condition improved following jejunostomy, plasma exchanges, large amount of fresh frozen plasma, IVIG, broad spectrum anti infection treatment and tofacitinib. The detailed changes of the patient's condition and treatment process are shown in Table 1. Based on this case and literature review, when JDM patients have gastrointestinal involvement, the use of high-dose glucocorticoids must be cautious, otherwise it may worsen gastrointestinal damage and affect disease prognosis. Perhaps biologics and JAKi may be better choices.

**Table 1:** Important events, laboratory tests and treatment forms before and after admission of the patient.

Time	Events	Key laboratory tests	Treatments
−6 m	Fatigue, weakness	Not done	Not done
−12 d	Hospitalization in local hosiptal with weakness, muscle pain, heliotrope rash, Gottron's papules	Positive with anti-NXP2 and ANA; MRI scans revealed inflammation and edema with muscle groups in both thighs; EMG indicate myogenic damage; elevated with CK, LDH, ALT and AST	High-dose methylprednisolone at 700 mg for three days; followed by a regimen of oral prednisone at 60 mg and methotrexate at 10 mg per week
1 d	Admission to our hospital; coughed while drinking liquid diet; heliotrope rash; Gottron's papules; BMI 13.3; CMAS 15/52	Normal with PCT, CRP and ESR	IV methylprednisolone
2 d	Vomiting; abdominal pain; paroxysmal coughing; chest pain; difficulty breathing; fever	Chest and abdominal CT scans revealed pulmonary inflammation and thickening of the right abdominal intestinal duct wall, elevated with PCT,CRP and ESR	Discontinued methotrexate; piperacillin tazobactam; fresh frozen plasma; IVIG
3 d	Transfer to PICU; recurrent fever; vomiting; abdominal pain; cough	Abdominal CT scan indicated duodenal dilation, intestinal and abdominal wall edema, perforation, elevated with PCT,CRP and ESR	Fresh frozen plasma; plasma exchange; IVIG; albumin; abrosia; gastrointestinal decompression; TPN
10 d	Recurrent fever; vomiting; abdominal pain; cough	Elevated with PCT,CRP and ESR	First surgery; vancomycin; meropenem; discontinued piperacillin tazobactam; TPN
14 d	Recurrent fever; vomiting; abdominal pain; cough	Elevated with PCT, CRP and ESR; ascites culture with candida tropicalis	Second surgery; discontinue glucocorticoid; voriconazole; amphotericin B; TPN
21 d	Recurrent fever; vomiting; abdominal pain; cough; GI bleeding, proteinuria, and hematuria	Elevated with PCT, CRP and ESR	Third surgery; jejunostomy; TPN; psychotherapy
58 d	Recurrent fever; abdominal pain; cough; BMI 11.1	Normal PCT; elevated with CRP and ESR	Distal jejunostomy pump milk; TPN
89 d	No fever; rash resolution; improvement of proximal muscle weakness	Normal with PCT, CRP, CK, LDH; elevated with ESR, AST and ALT	Tofacitinib; discontinued vancomycin, meropenem; TPN; psychotherapy and exercise rehabilitation
135 d	No fever; no rash; improvement of proximal muscle weakness	Normal with PCT, CRP, CK, LDH; elevated with ESR, AST and ALT	Discontinued voriconazole, amphotericin B; TPN; psychotherapy and exercise rehabilitation
192 d	Improvement of proximal muscle weakness	Normal with PCT, CRP, ESR, CK, LDH, AST and ALT	IV methylprednisolone; cyclophosphamide
230 d	Improvement of proximal muscle weakness	Normal with CRP, ESR, CK, LDH, AST and ALT	Repair the jejunostomy
264 d	Discharge, walk by herself; BMI 11.8; CMAS 42/52	Normal with CRP, ESR, CK, LDH, AST and ALT	Prednison, methotrexate

M, month; d, day; anti-NXP2, anti-nuclear matrix protein 2; ANA, antinuclear antibody; CT, computed tomography; MRI, magnetic resonance imaging; EMG, electromyography; CK, creatine kinase; LDH, lactate dehydrogenase; ALT, alanine aminotransferase; AST, aspartate aminotransferase; BMI, body mass index; CMAS, childhood myositis assessment scale; PCT, procalcitonin; CRP, C-reactive protein; ESR, red blood cell sedimentation rate; IV, intravenous infusion; IVIG, intravenous immunoglobulin; GI, gastrointestinal; TPN, total parenteral nutrition.

## Case description

**Figure 1:**
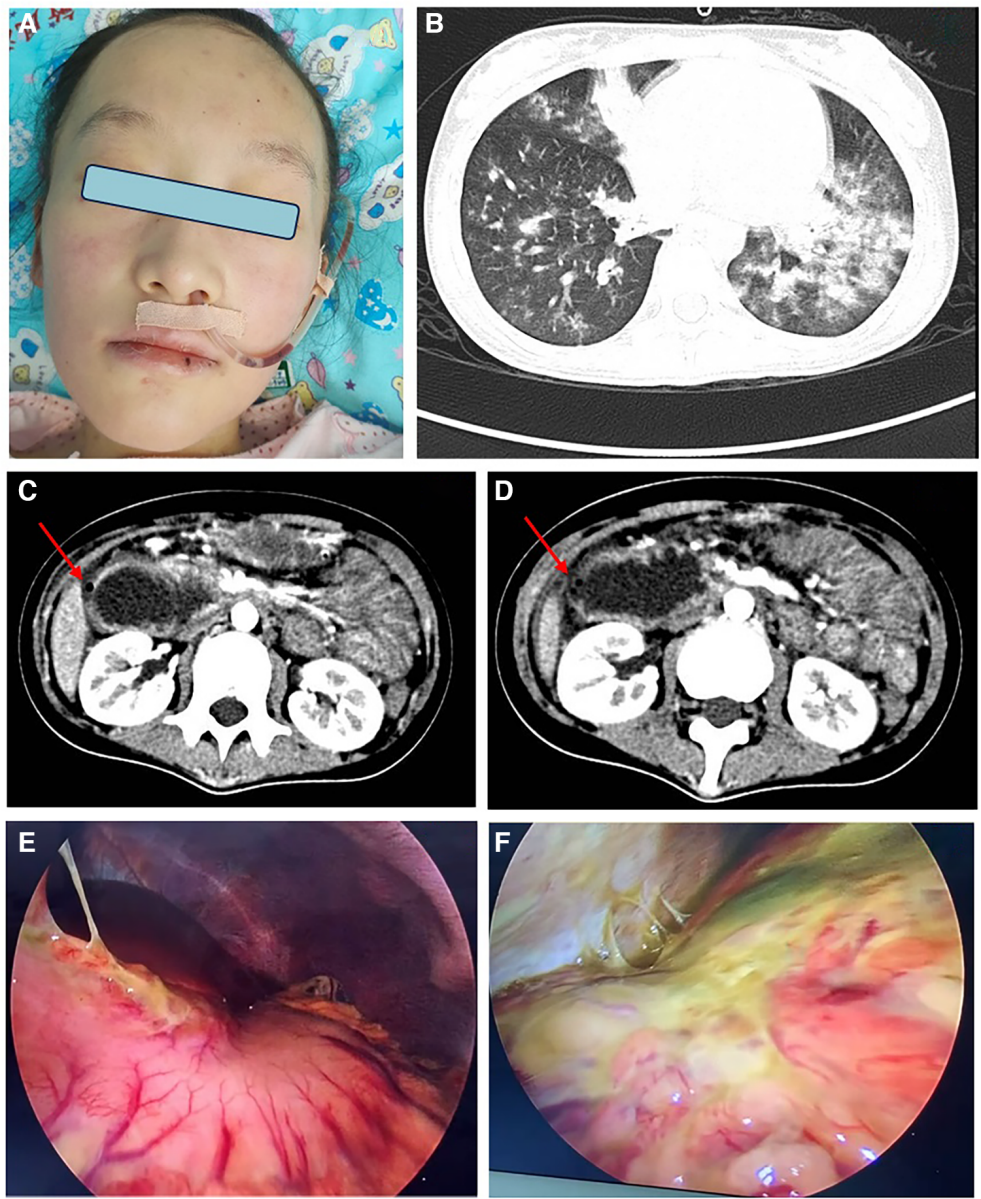
(**A**) Red patches and papules on the cheeks of this patient, via nasogastric decompression tube; (**B**) CT scan showed multiple patchy and patchy high-density shadows in both lungs; (**C, D**) CT scan of duodenal obstruction with perforation, intestinal wall edema is the site of perforation; (**E, F**) laparoscopic duodenal perforation with abdominal fluid accumulation and surface purulent secretion coverage.

This is a 12-year-old girl with no family history of rheumatic or genetic diseases. Six months ago, she presented with fatigue and weakness during daily activities, followed by myalgia and rashes on her upper eyelids, cheeks, neck, and the extensor aspects of her limbs (Figure 1A). Twelve days before admission to our hospital, she visited a local tertiary hospital, where she was diagnosed with JDM based on the presence of anti-NXP2 and a granular pattern of antinuclear antibody (ANA) at a titer of 1:100; Magnetic resonance imaging (MRI) scans revealed inflammation and edema affecting all muscle groups in both thighs; Electromyography (EMG) results indicated myogenic damage; Elevated levels of muscle enzymes were found, including creatine kinase (CK) at 131 U/L, lactate dehydrogenase (LDH) at 300.9 IU/L, alanine aminotransferase (ALT) at 60 U/L, and aspartate aminotransferase (AST) at 55 U/L. She was treated with high-dose methylprednisolone at 700 mg for three days, followed by oral prednisone at 60 mg daily and -methotrexate at 10 mg weekly. But the patient's symptoms did not show improvement and she transferred to our facility. Physical examination of the patient showed a height for 150centimeter, weight for 30 kilogram, and body mass index (BMI) of 13.3. There were some red rashes on both cheeks and right elbow, and muscle weakness in the proximal limbs. The score of childhood myositis assessment scale (CMAS) was 15/52.

Upon admission, the patient occasionally coughed while drinking a liquid diet. On the second night, she developed vomiting and abdominal pain, accompanied by paroxysmal coughing, chest pain, difficulty breathing, and a fever of 38 °C. Despite negative identification of pathogens, her chest and abdominal computed tomography (CT) scans revealed pulmonary inflammation and thickening of the right abdominal intestinal duct wall (Figure 1B). The patient was treated with piperacillin tazobactam for infection, fresh frozen plasma exchange, IVIG at a dosage of 2 g/kg but without improvement. Ten days later, repeat abdominal CT scan indicated duodenal dilation, intestinal and abdominal wall thickening with minimal gas accumulation, perforation in the posterior part of the bulb, multiple enlarged mesenteric lymph nodes, and significant pelvic fluid accumulation (Figures 1C,D). The patient underwent emergent surgery for perforation repair, but failed (Figures 1E,F). A leak was discovered at the surgical site, and new gastric and duodenal perforations were identified. Despite two additional repair surgeries for perforation were performed, but both failed. The patient continued to have recurrent fevers. Concurrently, the patient developed GI bleeding, proteinuria, and hematuria. Ultimately, the decision was made to abandon further attempts at perforation repair, and intestinal fistula was managed without glucocorticoids. She improved after the following treatments: six times of fresh frozen plasma exchange (initial parameters: blood pump speed 100 ml/min, exchange solution speed 750 ml/h, dehydration 0 ml/h), two times of blood purification, fresh frozen plasma support (total amount 19,300 ml), IVIG, tofacitinib at a dose of 5 mg twice daily via the distal ostomy site and anti-infections. The other maintenance therapy included total parenteral nutrition (TPN), fresh frozen plasma, albumin, electrolyte solution, psychotherapy and exercise rehabilitation. However, prolonged fasting and bed rest had a considerable impact on the patient's nutrition and physical fitness,leading to a weight loss from 30 kg to 25 kg. Enteral nutrition was initiated with formula milk through the distal fistula site. Re administering with IV methylprednisolone at a dosage of 1 mg/kg and cyclophosphamide at 5 mg/kg, which accelerated her improvement. By the eighth month of hospitalization, the patient underwent surgery to repair the jejunostomy. She recovered with a CMAS of 42/52 and normal blood assay indicators.

## Discussion

GI involvement and perforation represent grave complications in the context of both JDM and DM, carrying a particularly poor prognosis (13). The presence of anti-NXP2 antibodies in JDM patients is associated with a higher likelihood of severe GI complications (13). Research by Aouizerate et al. has shown that significant muscle ischemia and the presence of anti-NXP2 antibodies are linked to more severe JDM manifestations (3). A study conducted in 2021 by Xinning et al. reported 26 JDM patients with positive anti-NXP2, six experienced severe GI involvement, with five succumbing to their condition and one surviving following autologous stem cell transfer (ASCT) treatment. The study identified several risk factors for GI involvement and mortality, including severe muscle weakness, edema, skin ulcers, a BMI less than 15, and positivity for ANA (2). The initial symptoms of GI perforation in JDM typically include progressive abdominal pain and intermittent fever. The most common site for perforation is the duodenum, and this complication usually arises within the first 10 months following the diagnosis of JDM. In adults with DM, GI involvement often begins with abdominal pain and the presence of black stools, as noted in a retrospective study (2). Pathological examinations have revealed several key features of GI involvement in DM, such as deep vasculitis or vascular disease, necrosis of the intestinal smooth muscle, and serous calcification (13). In the case of NXP2-positive DM, micro-infarcts and MxA-positive inflammation are typical pathological findings (13). The phenomenon of epithelial-mesenchymal transition (EMT) has been observed, reflecting tissue repair and GI remodeling in the context of NXP2-positive DM. This process is consistent with the subacute nature of GI involvement, where the development of perforation can take weeks to months. Postoperative inflammation in these patients often persists and may recur (13). It is also important to consider the possibility of subclinical GI involvement occurring before the onset of symptoms. This highlights the need for enhanced sensitivity and timeliness in the screening process for GI complications in JDM patients. Early detection and intervention are crucial for improving patient outcomes and potentially preventing catastrophic GI events.

Here reported, the patient's experience from the onset of symptoms to a definitive diagnosis of JDM was a lengthy six-month period. Within two weeks of diagnosis, the patient was treated with high-dose methylprednisolone at 700 mg for three days, and GI symptoms emerged four days after the initiation of this treatment. Eleven days later, the patient suffered from multiple GI perforations in the duodenum and stomach, and attempts at surgical repair were unsuccessful. Several high-risk factors of developing GI complications were identified in this patient, including severe muscle weakness, a BMI below 15, edema of the abdominal wall, and a positive ANA and anti-NXP2 status. The use of high-dose steroid is a common therapeutic approach in refractory JDM but may also contribute to the occurrence of GI perforation. A review of forty-two randomized studies by Conn and Blitzer et al. indicated that glucocorticoids can elevate the risk of duodenal ulcers in patients who have been on the medication for over a month or have accumulated doses exceeding 1,000 mg of prednisone equivalent (7). In the context of JDM, this finding is particularly pertinent, as high-dose glucocorticoid therapy is often necessary to manage the disease effectively but may also heighten the risk of GI complications. Literature on JDM patients with GI perforation reveals a high mortality rate of 33.3% (11 out of 33 patients), underscoring the seriousness of this complication. Moreover, a significant proportion of these patients (36.4% or 12 out of 33) had received high-dose methylprednisolone therapy within a month prior to the development of GI perforation (2, 4–6, 10–12, 14–24). This temporal association suggests that high-dose methylprednisolone may not only be a risk factor for GI perforation but may also potentially accelerate its onset, beyond the impact of the primary disease. In the management of GI perforation, surgery remains the primary option, although the risk of leakage at the repair site is significant. During the acute phase of inflammation, fistulostomy surgery may offer a strategy to mitigate the risk of surgical leakage (4, 5, 15). Beyond surgical interventions, the treatment of JDM encompasses a range of therapies including glucocorticoids, immunosuppressants, IVIG, biological agents, JAKi, plasma exchange, and fresh frozen plasma support (4, 5, 14–24).

In the case reported here, the patient faced challenges with surgical leaks and the emergence of new perforations after GI perforation. Eventually, a jejunostomy was performed, and the patient was placed on fasting. The glucocorticoid was tapered and eventually discontinued after the second surgery. The treatment regimen was expanded to include broad-spectrum anti-infective measures, IVIG, plasma exchange, and fresh frozen plasma support. As the acute inflammatory phase improved, JAKi tofacitinib was introduced at a dose of 5 mg twice daily via the distal ostomy site, leading to a marked improvement in the patient's condition. JAKi represents an oral small molecule that inhibits tyrosine kinase phosphorylation within cells, offering the benefits of oral administration, convenience, and lack of immunogenicity. It has shown efficiency in treating refractory JDM and DM (25–27). According to the currently published literature, there are no reported cases of patients with gastrointestinal perforation in JDM treated with JAKi. However, there was a report of JAKi (tofacitinib) being used in JDM patients with gastrointestinal manifestations which reflect good effect (9). Biological agents, particularly anti-CD20 and TNF-α antagonists, have been considered for refractory JDM cases; however, their efficacy in the context of GI perforation remains a subject of debate and requires further evidence to substantiate their use.

The limitations of this article are that this is a retrospective case report, and the assessment indicators for treatment effects are not comprehensive, such as the comparison of cytokine levels before and after treatment. However, the case also provides an important reference for patients with refractory JDM with gastrointestinal perforation during the acute phase without the use of glucocorticoids. Drawing from the experience of this case and previous reports in the literature, further investigation is warranted to assess whether high-dose glucocorticoid therapy is essential following GI involvement during acute stage. JAKi may be a safe and effective option for treating refractory JDM in the acute phase. The patient here reported is very satisfied with the final treatment outcome.

## Conclusion

The involvement of the GI tract in JDM is a critical aspect of the disease that is associated with a poor prognosis and a significant risk of mortality, particularly following perforation. The use of high-dose glucocorticoids in JDM patients with GI involvement must be approached with caution, as there is a potential for these medications to exacerbate the condition, leading to increased risk of GI perforation and complicating treatment efforts.

In the context of refractory JDM with GI involvement, a treatment strategy that includes plasma exchange, fresh frozen plasma support, low-dose glucocorticoids, immunosuppressants and JAKi emerges as a relatively safer alternative. This approach may effectively manage the disease while minimizing the risk of further GI complications.

It is imperative that clinicians remain vigilant in monitoring JDM patients for signs of GI involvement and adjust treatment plans accordingly. The integration of JAKi and other targeted therapies into the management of JDM may offer a promising direction for improving outcomes in patients with GI complications, providing a more nuanced and personalized approach to care.

## Data Availability

The original contributions presented in the study are included in the article/Supplementary Material, further inquiries can be directed to the corresponding author.
